# The National Dental Association's Officers for 1907-1908

**Published:** 1907-08

**Authors:** 


### THE NATIONAL DENTAL ASSOCIATION'S OFFICERS FOR 1907-1908.

The National Dental Association at its 11th annual session, Minneapolis, July 31st., elected the following officers for the ensuing year: Pres. Wm. Carr, New York City; Vice-Pres. for the East, Wilbur F. Litch, Philadelphia, Pa.; Vice-Pres. for the South, J. P. Gray, Nashville, Tenn.; Vice-Pres. for the West, Alfred Owre, Minneapolis, Minn.; Cor. Sec'y, Burton Lee Thorpe, St. Louis, Mo.; Rec. Sec'y, Chas. S. Butler, Buffalo, N. Y.; Treas. A. S. Melendy, Knoxville, Tenn.

Executive Committee, New Members—L. Meisenburger, Buffalo, N. Y.; F. B. Kremer, Minneapolis, Minn.; M. F. Finley, Washington, D. C.

Executive Council—H. J. Burkhart, Chairman, Batavia. N. Y.; J. Y. Crawford, Nashville, Tenn.; A. H. Peck, Chicago, Ill.; F. O. Hetrick, Ottawa, Kansas; B. Holly Smith, Baltimore, Md.

Next place of meeting Boston, 1908.

